# IL-19 suppresses Hippo signaling via modulating YAP1 phosphorylation in osteoarthritis

**DOI:** 10.3389/fimmu.2025.1630253

**Published:** 2025-10-01

**Authors:** Jiezhong Deng, Jun Gu, Xiaoshan Gong, Hao Tang, Yuyi Cai, Yusheng Yang, Shiwu Dong, Chunrong Zhao

**Affiliations:** ^1^ Department of Orthopedics, Southwest Hospital, Army Medical University, Chongqing, China; ^2^ Department of Biomedical Materials Science, Army Medical University, Chongqing, China; ^3^ Department of Traumatic Orthopaedics (Joint Division), General Hospital of Xinjiang Military Region, Urumqi, China; ^4^ Department of Stomatology, Daping Hospital, Army Medical University, Chongqing, China

**Keywords:** IL-19, M2 macrophages, inflammation, apoptosis, OA, Hippo-yap

## Abstract

**Introduction:**

Osteoarthritis (OA) is one of the most prevalent chronic degenerative diseases, characterized by the progressive destruction of joints, which is primarily evidenced by alterations in the phenotype of chondrocytes, chondrocyte apoptosis, and the progressive fibrosis of cartilage. Interleukin19 (IL-19) is predominantly expressed and secreted by B cells, monocytes, and macrophages. Recent studies have demonstrated that IL-19 attenuated inflammatory responses and facilitated tissue repair. However, no reported studies have explored the effects of IL-19 on osteoarthritis.

**Method:**

We established an Interleukin 1β (IL-1β)-induced inflammation model and an anterior cruciate ligament transection (ACLT)-induced osteoarthritis model to validate the anti-inflammatory, anti-apoptotic, and osteoarthritis-delaying effects of IL-19.

**Results:**

This study found an elevated expression of IL-19 in the joints of OA mice and confirmed that the IL-19 in these joints primarily derives from synovial M2 macrophages. Additionally, we found that IL-19 mitigates IL-1β-induced osteoarthritis by inhibiting the Hippo signaling pathway and the phosphorylation of the YAP1 (Yes-associated protein 1) protein.

**Conclusion:**

IL-19 represses the inflammatory response and apoptosis of IL-1βw-induced chondrocytes, thereby helping to delay the progression of osteoarthritis.

## Introduction

Osteoarthritis (OA) is characterized by cartilage degeneration, synovitis, changes in subchondral bone, and osteophyte formation, all of which contribute to the loss of joint function (1). Clinically, patients exhibit symptoms such as chronic pain, joint instability, stiffness, joint deformation, and reduced joint space (2). Among these, cartilage degeneration is the most critical pathological feature leading to joint function damage. This degeneration is mainly manifested by changes in chondrocyte phenotype, chondrocyte hypertrophy and apoptosis, as well as progressive fibrosis of cartilage (3, 4). As the only cellular component in articular cartilage, chondrocytes play a pivotal role in sensing changes in the joint microenvironment, regulating the synthesis and degradation of cartilage matrix, and maintaining the structural integrity of cartilage (5). The apoptosis of chondrocytes disrupts the delicate balance between cartilage matrix synthesis and degradation; which promotes further chondrocyte apoptosis, thereby accelerating the progression of OA (6). Consequently, chondrocyte apoptosis has become a key part of the research on the pathogenesis of OA.

OA patients often experience significant inflammatory reactions in their joints, manifested as swelling and pain. Interleukin 1 beta (IL-1β) and Tumor necrosis factor-alpha (TNF-α) are detectable in the synovial fluid of OA patients, and the intensity of inflammation is positively correlated with the degree of cartilage degeneration (7). These factors also drive chondrocytes to produce matrix-degrading enzymes, including Matrix metalloproteinases (MMPs) and A disintegrin and metalloproteinase with thrombospondin motifs (ADAMTS), facilitating the degradation of the cartilage matrix (8, 9).

Currently, the primary pharmacological treatment for OA includes the use of anti-inflammatory drugs, such as nonsteroidal anti-inflammatory drugs (NSAIDs) (10). These drugs can help alleviate OA symptoms to some extent; however, they do not address the underlying cause of OA and exhibit severe side effects (11). Therefore, there is an urgent need for anti-inflammatory and anti-apoptotic drugs with fewer adverse reactions to improve the clinical efficacy of OA patients. Interleukin 19 (IL-19) is a member of the Interleukin10 (IL-10) cytokine family, which is primarily produced and secreted by B cells, monocytes, and macrophages (12). Recent studies have shown that IL-19 can mitigate inflammatory responses and facilitate tissue repair. Research has shown that the expression of IL-19 is upregulated in infected human macrophages, which suppresses inflammatory responses by inhibiting the production of TNF-α and IL-6 (13). Furthermore, the expression of IL-6 and TNF-α is markedly increased in activated microglia of IL-19 knockout mice (14). On the other hand, IL-19 is also essential for promoting cell proliferation and resisting apoptosis. Research indicates that overexpression of IL-19 promotes proliferation and migration in non-invasive 67NR cancer cells by activating the JAK1/STAT3 signaling pathway (15). It was also found that IL-19 exerts anti-apoptotic effects by reducing reactive oxygen species (ROS) in human vascular smooth muscle cells (16). However, little is known about the function of IL-19 in OA.

It has been indicated that enhanced M2 macrophage polarization can stimulate the secretion of anti-inflammatory cytokines, such as IL-10, TGF-β, IL-4, and IL-13, which significantly inhibit the progression of OA (17). In addition to suppressing synovial inflammation via anti-inflammatory factors, M2 macrophages also secrete exosomes, which exert paracrine effects on chondrocytes and promote cartilage tissue repair and regeneration (18). Moreover, treatment with dexamethasone enhanced M2 macrophage polarization and increased the secretion of IL-19, IL-10, and other anti-inflammatory cytokines in children with lupus nephritis, facilitating the repair of tissue damage (19).

The impact of IL-19 on osteoarthritis remains unclear. This study intends to reveal the anti-inflammatory, anti-apoptotic, and osteoarthritis-delaying effects of IL-19 by IL-1β-induced inflammation model and anterior cruciate ligament transection (ACLT)-induced osteoarthritis model. Moreover, this study investigates the source of IL-19 within the joint and assesses the regulatory influence on the Hippo-YAP signaling pathway.

## Materials and methods

### Reagents

Cell counting kit-8 and type II collagenase were purchased from Beyotime (Shanghai, China);Tunel staining kit was purchased from Beyotime; IL-1β, IL-19, and IL-20Rβ neutralizing antibody were purchased from MCE (New Jersey, USA); IL-4, IL-13, and M-CSF were purchased from Peprotech (New Jersey, USA); Primary antibodies against COX-2 (27308-1-AP), iNOS (22226-1-AP), Col II (28459-1-AP), ACAN (13880-1-AP), MMP13 (18165-1-AP), TNF-α (26162-1-AP), and GAPDH (10494-1-AP) were purchased from Proteintech (Wuhan, China); IL6 (MB9297), ADAMTS5 (BS74041) and β-actin (AP0060) were purchased from Bioworld (MN, USA); Caspase3 (AFRM80113), Caspase7 (AFRM80577), Bax (AFRM9175) and Bcl-2 (AFRM9319) antibodies were obtained from AiFang biological (Hunan, China); CD206 (24595), MOB1A (13730), LATS1 (3477), TEAD1 (12292), p-YAP1 (13008), and YAP1 (14074) were purchased from CST (MA, USA); Enzyme-linked immunosorbent assay (ELISA) kits to detect IL-19 were purchased from Biosharp (Anhui, China).

### Isolation and culture of primary mouse articular chondrocytes

The primary chondrocytes were harvested from neonatal (0–7 days of age) C57BL/6 mice. The hips joint was dislocated using scalpels and forceps to keep the femoral head intact. Then removed skin and soft tissues from the hindlimbs. The cartilage cap of each femoral head and articular cartilage from the femoral condyles and tibial plateau were cut into fragments finely. The cartilage fragments were incubated in 5 ml trypsin (Biosharp, China) for 10 min at 37 °C. After removing the trypsin solution, the cartilage fragments were covered with type IV collagenase (Sigma, USA) in DMEM (Hyclone, USA) for 6–8 hours. Then the sample was filtered with a 40 μm cell strainer (Biosharp, China) and the obtained suspension were centrifuged at 2000 rpm for 5 min. The obtained cells were cultured in DMEM with 10% FBS (Hyclone, USA) and 1% penicillin/streptomycin (Thermo, USA) in a humidified incubator with 5% CO_2_ at 37 °C. Cells were passaged with 0.25% trypsin for 1 min at room temperature until the cells reached 90% confluence. The second generation of cells were used for the following experiments.

### Isolation and purification of primary BMMs cells

Four-week-old C57BL/6 mice were purchased from the animal experiment center of Army medical university. The tibia and femur were dissected and removed in a clean bench, then the bone marrow cavity was continuously rinsed with DMEM medium until the red bone marrow was completely washed clean. The bone marrow was mixed by pipette aspiration to form individual cells. The solution was filtered through 70 μm cell (Biosharp, China) sieve and centrifuged at 1000 rpm for 5 min. The supernatant was discarded, and 4 mL of red blood cell lysate was added. The mixture was centrifuged and the supernatant was discarded. The cells were resuspended in α-MEM (Hyclone, USA) and inoculated into T25 cell flasks. After 72 hours, the cells were observed, washed three times with PBS (Biosharp, China) to remove non-adherent cells, and then conditional culture medium was added for further cultivation to induce macrophage differentiation.

### TUNEL

Chondrocytes were seeded in 6 well plates and treated with or without IL-19 (50 ng/ml) for 24 hours. After that, cells were stimulated with or without IL-1β (10 ng/ml) for another 24 hours. Cells were treated with 4% paraformaldehyde (Beyotime, China) for 15 min. After fixation, cells were permeabilized with 0.1% Triton X-100 (Beyotime, China) for 10 min at room temperature. Next, cells were washed with PBS and stained with a TUNEL staining kit according to the manufacturer’s instructions. Nikon ECLIPSE Ti microscope was used to observe apoptotic cells in each group. Finally, images were acquired with a confocal laser scanning microscope (Leica Microsystems, Germany).

### CCK-8

Primary chondrocytes were divided into 6 groups in a 96 well plate. After adhesion, IL-19 (0, 10, 20, 50, 100, and 200 ng/mL) was added with or without IL-1β (10 ng/ml). The plate was then placed in an incubator for 24 hours. The supernatant was subsequently discarded, and 100 μl of CCK-8 working solution (prepared by combining 10 μl of CCK-8 reagent with 90 μl of cartilage culture medium) was added to each well and incubated for 4 hours. Absorbance at 450 nm was measured with a Multiskan FC Microplate Photometer (Thermo Fisher Scientific, Waltham, USA).

### ELISA

IL-19 standard solution, washing working solution, biotin-labeled antibody working solution, and horseradish-peroxidase-labeled avidin working solution were prepared according to the instructions. The collected orbital blood was allowed to stand at room temperature for 2 hours and then centrifuged at 1000 rpm for 15 min at room temperature. The chondrocyte supernatant was centrifuged at 2000 rpm for 3 min at room temperature, and the supernatant was retained. Wells were assigned to standard and test sample groups. 100 μl of the corresponding solution was added to each well, gently shaken to mix, covered with sealing film, and incubated at 37 °C for 2 hours. The supernatant was discarded, 100 μl of biotin-labeled antibody was added, the plate was covered with a sticker, and incubation was carried out at 37 °C for 1 hour. The supernatant was discarded, and the wells were washed three times with PBS. 100 μl of horseradish-peroxidase-labeled solution was added, the plate was covered with a sticker, and incubation was performed at 37 °C for 1 hour. The supernatant was discarded, and the wells were washed five times. 90 μl of substrate was added, and the plate was incubated in the dark at 37 °C for 15–30 min. Finally, 90 μl of stop solution was added. The absorbance at 450 nm was measured with a Multiskan FC Microplate Photometer.

### Western blot and Co-IP

Equal amounts of total protein (30 μg) extracted from chondrocytes were separated by 10% SDS-PAGE gels and then transferred to PVDF membranes (Bio-Rad, USA) at a constant current (300 mA) for 2 hours. The membrane was blocked with 5% BSA for 2 hours before incubation with the primary antibodies overnight at 4 °C. Subsequently, the membranes were incubated with the secondary antibodies for 2 hours at room temperature. Eventually, the blots were visualized by Enhanced Chemiluminescence (ECL) kit (Biosharp, China), and the intensities of these blots were quantified using Image Lab 3.0 software (Bio-Rad). For Co-IP, whole-cell extracts were prepared by using lysis buffer after transfection and were incubated with the YAP1 antibody overnight at 4 °C. Protein A&G beads were added and the incubation was continued for 4 hours at 4 °C. Coprecipitated proteins were washed, eluted with SDS-loading buffer at 95 °C for 5 min, and then subjected to western blot analyses. The Co-IP experiment used ACE’s IP kit, and all reagents and steps were completed according to the instructions of the kit.

### RT−qPCR

Chondrocytes were washed with cold PBS and incubated with Trizol reagent (Thermo, USA) to extract total RNA. The total RNA was quantified with a spectrophotometer at 260 nm (Thermo Scientific NanoDrop 2000), and the ratio of the absorbance at A260/A280 was used to evaluate the purity of the RNA. The cDNA was synthesized using 1 µg RNA with a PrimeScript™ RT Master Mix (Takara, Japan). Then, cDNA was subjected to RT−qPCR analysis with SYBR^®^Fast qPCR Mix (Takara, Japan) by CFX96 Real−Time PCR system (Bio−Rad) under conditions of 94 °C for 30 sec, followed by 40 cycles at 95 °C for 5 sec and 60 °C for 10 sec, and finally the dissociation curve of each primer pair was analyzed to determine primer specificity. Target mRNA levels were normalized to the *GAPDH* level. The primers are shown in **Table 1**.

**Table 1:** Sequences of primers used in quantitative polymerase chain reaction analysis.

Gene	Sequences (5’→3’)
MMP13	Forward CACAGTTGACAGGCTCCGAGAA Reverse CCACATCAGGCACTCCACATCT
ADAMTS5	Forward TCAGACTTGGTGGAGGCGTAGG Reverse AGGCGGATGTGGTTCTCAATGC
GAPDH	Forward GTGGCAAAGTGGAGATTGTTG Reverse CGTTGAATTTGCCGTGAGTG
Col II	Forward TGGAGCAGCAAGAGCAAGGAA Reverse TCAGTGGACAGTAGACGGAGGA
Aggrecan	Forward AAGAATCAAGTGGAGCCGTGTT Reverse CAGAGTCATTGGAGCGAAGGTT
Bax	Forward TGGAGCTGCAGAGGATGATTG Reverse GGGGTCCCGAAGTAGGAGAG
Bcl-2	Forward GGATAACGGAGGCTGGGATG Reverse GCTGAGCAGGGTCTTCAGAG
Caspase-3	Forward AGCTTGGAACGGTACGCTAA Reverse GAGTCCACTGACTTGCTCCC
Caspase-7	Forward CCGTGGGAACGATGACCG Reverse GCGGTACAGATAAGTGGGCA

### Immunofluorescence

Chondrocytes were seeded in 6-well plates and treated with IL-19 (50 ng/ml) for 24 hours. Then, cells were stimulated with or without IL-1β (10 ng/ml) for another 24 hours. Cells were treated with 4% paraformaldehyde for 15 min, 0.1% Triton X-100 for 15 min, and goat serum for 1 hour at room temperature. After that, cells were incubated with the primary antibody YAP-1 (1:200) at 4 °C overnight. After washing with PBS, cells were incubated with Alexa Fluor^®^488-labeled goat anti-rabbit IgG (H+L) secondary antibody (1:400) for 1 hour in darkness. Finally, cells were washed with PBS and incubation with DAPI (Beyotime, China) for 2 min. Images were acquired with a confocal laser scanning microscope (Zeiss, Germany).

### Proteomics and phosphorylation omics

Protein was extracted from chondrocytes, and its concentration was determined with a BCA protein assay kit (Beyotime, China). 20 µg of protein from each sample was mixed with sample buffer and boiled for 5 min. A 12.5% SDS-PAGE gel was prepared, the samples were loaded, and electrophoresis was performed at a constant current of 14 mA for 90 min. Protein bands were visualized by Coomassie Brilliant Blue R-250 staining, enabling the separation of phosphorylated and non-phosphorylated proteins. 30 µg of protein was added to 200 µl SDT buffer and mixed thoroughly. Detergent, DTT, and other low-molecular-weight components were removed by repeated ultrafiltration with UA buffer. 100 µl iodoacetamide was added to block cysteine residues, mixed gently, and the sample was incubated in the dark for 30 min. The sample was washed twice with 100 µl of 25 mM NH_4_HCO_3_ solution. Finally, 4 µg/µl trypsin and 40 µl NH_4_HCO_3_ solution were added, mixed thoroughly, and digestion was carried out overnight at 37 °C; the resulting filtrate was collected as the peptide fraction. The peptide fraction was desalted on an Empire™ SPE C18 cartridge, concentrated under vacuum, and reconstituted in 40 µl of 0.1% formic acid.

Peptide content was estimated from the UV absorbance at 280 nm. The sample was reconstituted in 1.4 ml pre-chilled IAP buffer. TiO_2_ beads were added to the mixture, shaken for 40 min, centrifuged, and the supernatant was discarded. The beads were transferred into a stoppered tip, washed three times with washing buffer 1, followed by three washes with washing buffer 2. Phosphopeptides were eluted with elution buffer, concentrated under vacuum, and redissolved in 20 µl of 0.1% formic acid.

LC-MS/MS analysis was performed on a timsTOF Pro mass spectrometer (Bruker) coupled to a NanoElute system (Bruker Daltonics) for 60 min. Peptides were loaded onto a C18 reverse-phase analytical column in buffer A (0.1% formic acid) and separated using a gradient of buffer B (84% acetonitrile, 0.1% formic acid) at a flow rate of 300 nl min^-1^. The mass spectrometer was operated in positive-ion mode.

### Animal models

8-week-old male C57BL/6 mice (n=45) purchased from animal center of the Army Medical University were kept under standard animal room conditions in a 12-hours light/dark cycle and fed a standard laboratory diet. All experimental procedures were approved by the Animal Care Committee of the Army Medical University. Mice were randomly grouped as follows: the sham group, the OA group, and the OA+IL-19 group (n=15/group). After the mice were anesthetized, the OA model was established via transection of anterior cruciate ligament (ACLT). After surgery, mice from the sham group and the OA group were administered of 3 μl PBS, while the OA+IL-19 group received 100 ng IL-19 dissolved in 3 μl PBS via intra-articular injection through a medial parapatellar approach every 3 days. A total of 5 mice from each group were sacrificed at 2, 4, and 8 weeks after surgery.

### Specimen preparation and histological analysis

Tibia and femur tissues were separated and fixed in 4% paraformaldehyde for at least 48 hours and then decalcified in 10% EDTA (pH 7.3) for 3 weeks. The specimens were embedded in paraffin and cut into 4 µm sections for immunofluorescence staining with IL-19, CD206, BAX, iNOS, TNF-α, and MMP13.

### Immunoprecipitation and mass spectrometry analysis

Harvest cells, add an appropriate amount of cell IP lysis buffer (containing protease inhibitors), lyse on ice or at 4 °C for 30 minutes, centrifuge at 12000 rpm for 30 min, and then take the supernatant. Take a small amount of lysis buffer for western blot analysis. Add 1 μg of YAP1 antibody and 10-50 μl of protein A/G-beads to the remaining lysis buffer, and slowly shake at 4 °C for overnight incubation. After immunoprecipitation reaction, centrifuge at 3000 rpm for 5 min at 4 °C, and centrifuge protein A/G-beads to the bottom of the tube. Carefully aspirate the supernatant and wash protein A/G-beads 3-4 times with 1 ml lysis buffer, add 15 μl of 2 × SDS sample buffer and boil in boiling water for 10 min. Finally, perform mass spectrometry analysis.

### ChIP-seq and ChIP-PCR

Cells were cultivated in a 10 cm dish with 10 ml of culture medium. Formaldehyde was added to the medium and gently mixed to a final concentration of 1%. The dish was immediately incubated at 37 °C for 10 min to allow cross-linking between the target protein and its cognate genomic DNA. 1.1 ml of 10 × Glycine Solution was added and mixed gently, followed by a 5 min incubation at room temperature. Medium was removed on ice, and the cells were washed twice with 5–10 ml of ice-cold PBS containing 1 mM PMSF. Cells were scraped off in 1 ml of ice-cold PBS containing 1 mM PMSF and collected in centrifuge tubes. Cells were counted and aliquoted at ~1 × 10^6^ cells per tube. After centrifugation at 4 °C, 1000 rpm for 3 min, supernatants were discarded. Cell pellets were resuspended in 0.2 ml SDS Lysis Buffer containing 1 mM PMSF and lysed on ice for 10 min. Genomic DNA was fragmented to 200–1000 bp by sonication. After centrifugation at 4 °C, 12 000 rpm for 5 min, supernatants (~0.2 ml) were transferred to 2 ml tubes kept on ice. Samples were diluted 1:10 by addition of 1.8 ml ChIP Dilution Buffer containing 1 mM PMSF.

20 μl of each diluted sample was set aside as “input” for later analysis. 70 μl of Protein A+G Agarose/Salmon Sperm DNA was added to the remaining sample, which was then rotated slowly at 4 °C for 30 min. After centrifugation at 4 °C, 1000 rpm for 1 min, supernatants were transferred to new 2 ml tubes. An appropriate amount of YAP1 antibody was added, and the tubes were rotated slowly at 4 °C overnight. 60 μl of Protein A+G Agarose/Salmon Sperm DNA was added, followed by slow rotation at 4 °C for 60 min. After centrifugation at 4 °C, 1000 rpm for 1 min, the beads were washed sequentially for 5 min each with Low Salt Immune Complex Wash Buffer, High Salt Immune Complex Wash Buffer, LiCl Immune Complex Wash Buffer, and finally twice with TE Buffer. The washed precipitate was retained for DNA sequencing and PCR.

### Dual luciferase assay

A database was used to predict the binding sites within the promoter sequences of Smad2 and TEAD1. Transcription-factor overexpression vectors, as well as wild-type (WT) and mutant (mut) dual-luciferase reporter vectors carrying the Smad2 promoter, were designed and constructed. The WT and mut dual-luciferase vectors together with the TEAD1 overexpression vector were then co-transfected into 293T cells according to the designated groups.

### DNA pull-down

A DNA probe for Smad2 was synthesized and biotinylated. The biotin-labeled Smad2 promoter probe was amplified by PCR, and the PCR products were resolved by agarose gel electrophoresis, from which the Smad2 promoter probe was recovered by gel excision. The biotinylated DNA probe was immobilized onto streptavidin magnetic beads. Nuclear proteins were extracted and added to the saturated bead suspension containing the bound probe. The SDS–magnetic-bead–probe–protein mixture was resuspended and incubated, after which the supernatant was collected for western blot.

### Statistics analysis

All experimental data were analyzed using GraphPad Prism (version 9.0) and were shown as mean ± standard error of the mean (SEM). All experiments were independently repeated three times. Comparisons between two groups were analyzed by using independent unpaired two-tailed Student’s t-tests and comparisons among more than three groups were analyzed by using ANOVA test. P < 0.05 was considered to indicate a statistically significant difference.

## Results

### IL-19 is upregulated in M2 macrophages in OA mice

**Figure 1:**
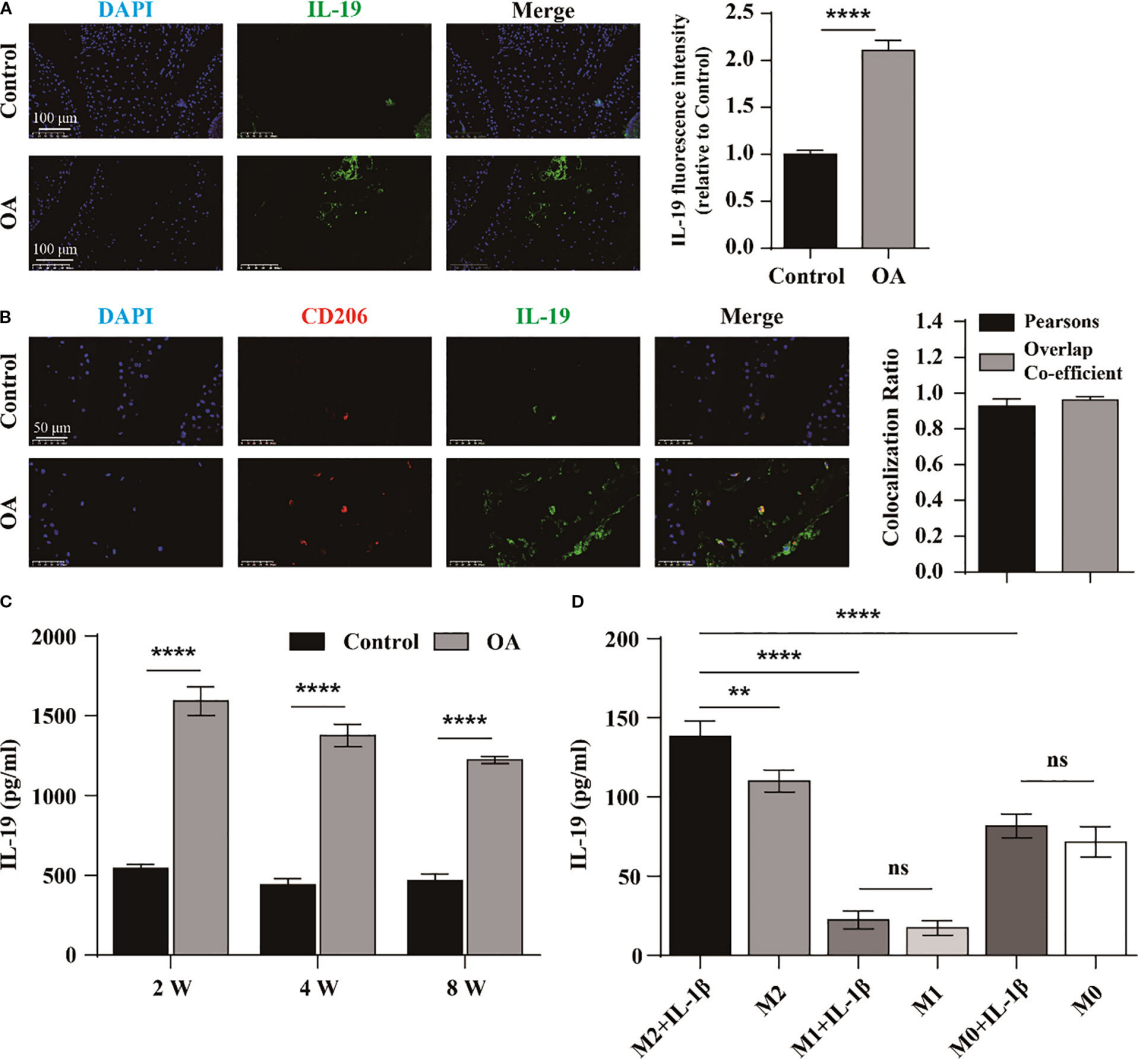
IL-19 is upregulated in M2 macrophages in OA mice. **(A)** Immunofluorescence staining and immunofluorescence intensity statistical analysis of IL-19 in mouse joint synovium. scale bar: 100 μm. **(B)** Immunofluorescence staining and co-localization analysis of IL-19 and CD206 in mouse joint synovium. scale bar: 50 μm. **(C)** ELISA detection of IL-19 concentration in mouse serum. **(D)** ELISA detection of IL-19 concentration in various groups of cell supernatant. The data in each panel represent the means ± SD and p values were obtained by Student’s two-tailed unpaired t test **(A, C)** and ANOVA test **(D)**, ** p < 0.01, *** p < 0.001, **** p < 0.0001, ns: non-significant.

To investigate the relationship between IL-19 and OA, we first measured the average fluorescence intensity of IL-19 in the synovium tissue of both the control group and the OA group of mice by immunofluorescence staining. The results demonstrated that the average fluorescence intensity of IL-19 was significantly higher in the OA group than that in the control group (**Figure 1A**). It indicates a potential association between IL-19 and the progression of osteoarthritis. Then, we detected the concentration of IL-19 in the serum of both control and OA mice at 2, 4, and 8 weeks. The results revealed that the concentration of IL-19 was significantly elevated in the serum of OA mice compared to that of the control group (**Figure 1C**). To explore the potential source of IL-19 within the OA joints, an osteoarthritis model in mice was established by ACLT technology for 4 weeks, and immunofluorescence staining was used to visualize the expression of IL-19 and CD206 (**Figure 1B**). Additionally, we performed co-localization analysis and found a strong correlation (Pearson’s correlation coefficient > 0.8) between IL-19 and CD206 within the OA joints (**Figure 1B**). As CD206 is a canonical marker for M2 macrophages, we hypothesize that intra-articular IL-19 may originate from synovial M2 macrophages. To validate it, we induced M1 polarization with LPS and IFN-γ, and M2 polarization with IL-4 and IL-10. To simulate the inflammatory conditions of OA *in vitro*, macrophages were treated with 10 ng/ml IL-1β for 24 hours. We measured the concentration of IL-19 in the supernatant from each group of cells by ELISA. The results indicated that M2 macrophages exhibited a significantly greater capacity for IL-19 secretion compared to M0 and M1 macrophages, and was further enhanced upon IL-1β stimulation (**Figure 1D**). These findings confirm that M2 macrophages are the primary cell tyle responsible for secreting IL-19 in the OA joints.

### M2 macrophages derived IL-19 inhibits IL-1β induced degradation of extracellular matrix of chondrocytes

**Figure 2:**
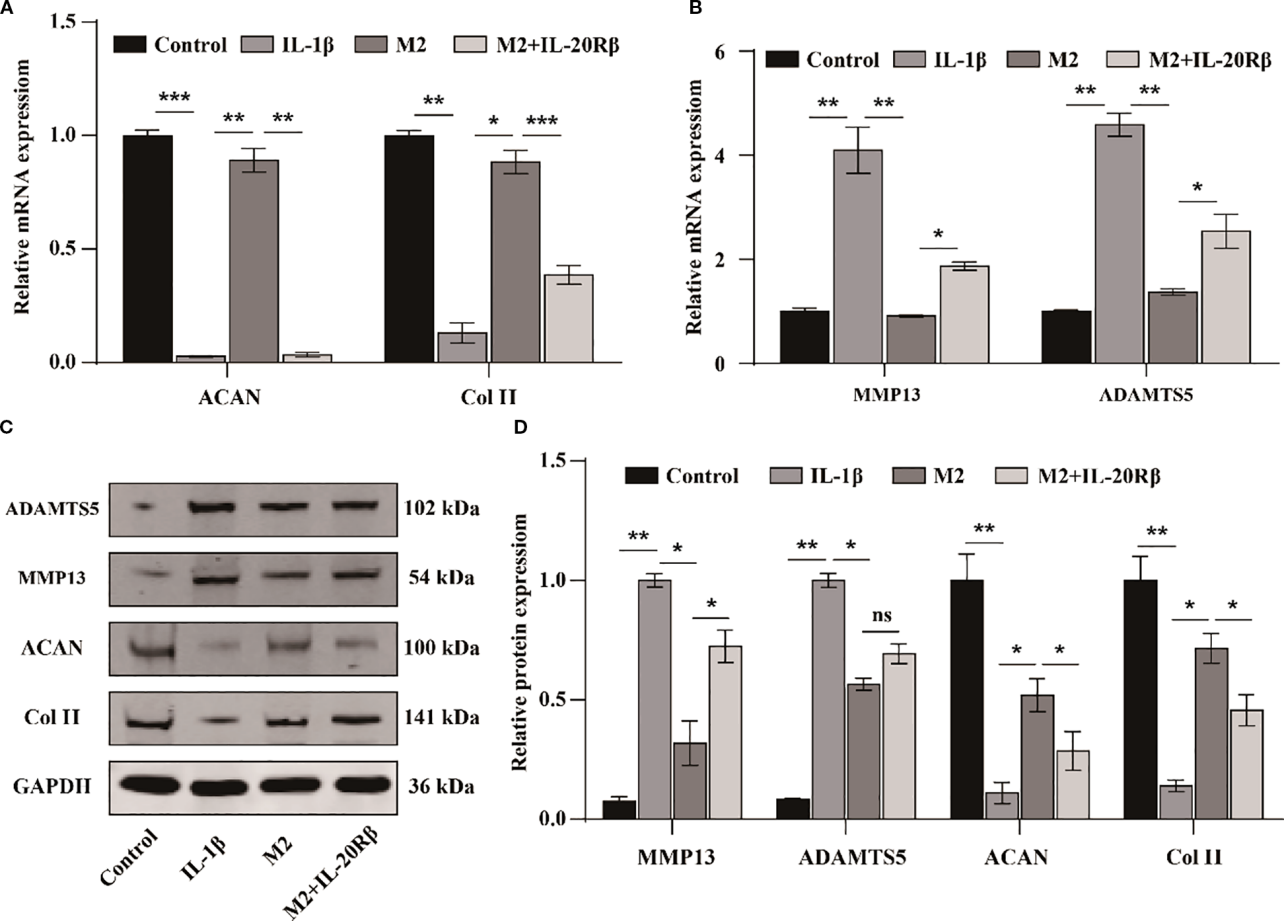
M2 macrophages derived IL-19 inhibits IL-1β induced degradation of extracellular matrix of chondrocytes. **(A)** QPCR detection of mRNA expression of ACAN and Col II. **(B)** QPCR detection of mRNA expression of MMP13 and ADAMTS5. **(C)** Western blot detection of protein expression of ACAN, Col II, MMP13 and ADAMTS5. **(D)** Western blot band semi quantitative analysis. The data in each panel represent the means ± SD and p values were obtained by ANOVA test, * p < 0.05, ** p < 0.01, *** p < 0.001, ns: non-significant.

Recent studies have demonstrated that blocking IL-20Rβ with a neutralizing antibody significantly inhibits IL-19 signal transduction at high concentrations (20). To further confirm the involvement of IL-19 derived from M2 macrophages in the progression of OA, we treated chondrocytes with various conditions: DMEM medium (Control), DMEM medium supplemented with IL-1β (10 ng/ml), M2 conditioned medium with IL-1β, and M2 conditioned medium with IL-1β plus anti-IL-20Rβ (100 ng/ml). Following treatments, the expressions of cartilage matrix synthesis and degradation-related genes were assessed by western blot and qPCR. The qPCR results indicated that treatment with IL-1β significantly suppressed the expression of ACAN and Col II (**Figure 2A**), while concurrently promoted the expression of MMP13 and ADAMTS5 (**Figure 2B**). In contrast, M2 conditioned medium markedly enhanced the expression of ACAN and Col II (**Figure 2A**) and inhibited the levels of ADAMTS5 and MMP13 compared to the IL-1β group (**Figure 2B**). However, the beneficial effects of M2 conditioned medium on ACAN and Col II expression were obstructed following treatment with the neutralizing antibody against IL-20Rβ (**Figure 2A**), and the inhibitory effect on MMP13 and ADAMTS5 were diminished (**Figure 2B**). Correspondingly, western blot analysis revealed similar changes in these gene expressions (**Figures 2C, D**). Collectively, these results suggest that M2 macrophages may inhibit the degradation of the extracellular matrix in chondrocytes via the IL-19/IL-20Rβ signaling pathway.

### The inhibitory effect of IL-19 on the apoptosis of chondrocyte

**Figure 3:**
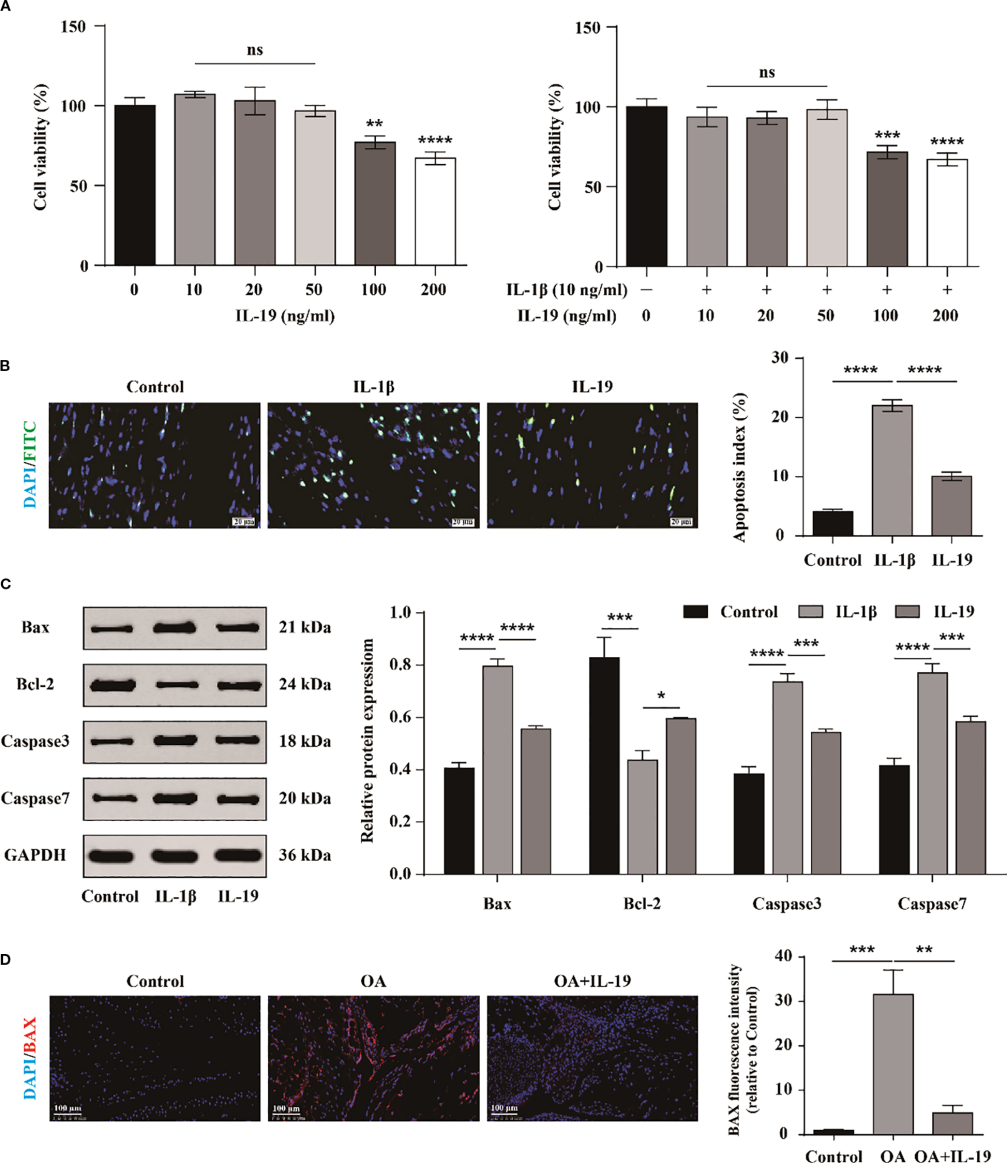
The inhibitory effect of IL-19 on the apoptosis of chondrocyte. **(A)** CCK-8 detection of the growth inhibitory effect of different concentrations of IL-19 on chondrocytes treated with or without IL-1β. **(B)** Tunel assay for detecting cell apoptosis and apoptosis index statistical analysis. **(C)** Western blot detection of protein expression of Bax, Bcl-2, Caspase3, and Caspase7. **(D)** Immunofluorescence staining and immunofluorescence intensity statistical analysis of Bax in mouse joint synovium. scale bar: 100 μm. The data in each panel represent the means ± SD and p values were obtained by ANOVA test, * p < 0.05, ** p < 0.01, *** p < 0.001, **** p < 0.0001, ns: non-significant.

To investigate the cytotoxic effects of IL-19 on chondrocyte growth and to establish the optimal treatment concentration, chondrocytes were exposed to various concentrations of IL-19 (0, 10, 20, 50, 100, and 200 ng/mL) for a duration of 24 hours. The growth and proliferation of chondrocytes were assessed by the CCK-8 assay. The results indicated that IL-19 at concentrations of 10, 20, and 50 ng/ml did not exhibit significant cytotoxicity on primary mouse chondrocytes; however, higher concentrations of IL-19 (100 and 200 ng/ml) reduced the cellular activity of chondrocytes (**Figure 3A**). To further explore the influence of IL-19 on IL-1β-induced cytotoxicity, chondrocytes were pre-treated with 10 ng/ml IL-1β for 24 hours, then different concentrations of IL-19 (0, 10, 20, 50, 100, and 200 ng/ml) were used to intervene in chondrocytes for 24 hours. CCK-8 results were consistent with that was stimulated with IL-19 alone (**Figure 3A**). These findings demonstrated that chondrocyte proliferation activity remained largely unchanged with lower concentrations of IL-19, leading us to proceed with subsequent cellular experiments using 50 ng/ml of IL-19. To further validate the direct effect of IL-19, we treated chondrocytes with DMEM medium, DMEM medium supplemented with IL-1β (10 ng/ml), and DMEM with IL-1β plus IL-19 (50 ng/ml), labeling them as Control, IL-1β, and IL-19 respectively. Firstly, apoptosis in chondrocytes across each group was assessed via TUNEL staining. Statistical analysis revealed that IL-1β significantly enhanced chondrocyte apoptosis, while the application of IL-19 markedly reduced the percentage of apoptotic cells (**Figure 3B**). Moreover, western blot analysis demonstrated that treatment with IL-19 significantly inhibited the protein expression of apoptosis promoting factors Bax and Caspase-3/7, while significantly promoted the levels of the anti-apoptotic factor Bcl-2 (**Figure 3C**). To further examine the effects of IL-19 on OA cartilage *in vivo*, an OA mouse model was established through ACLT, followed by IL-19 injection into the joint cavity. Samples were taken 4 weeks later and results of immunofluorescence staining for BAX showed that IL-19 treatment significantly mitigated the positive signals of the pro-apoptotic factor BAX (**Figure 3D**). Together, these findings underscore the potential of IL-19 in inhibiting chondrocyte apoptosis in the context of osteoarthritis.

### IL-19 inhibits the inflammatory response of chondrocytes

**Figure 4:**
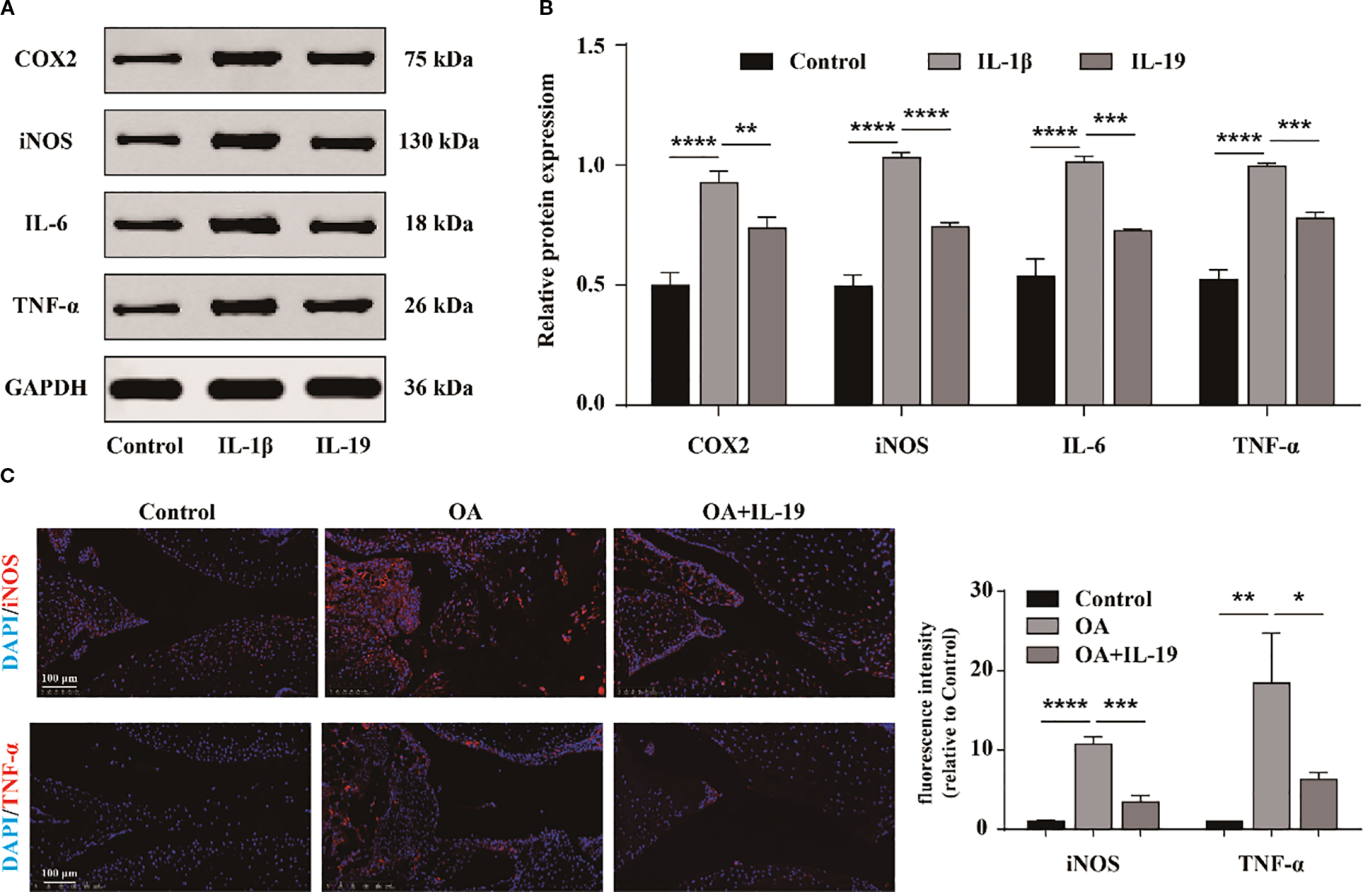
IL-19 inhibits the inflammatory response of chondrocytes. **(A)** Western blot imaging of TNF-α, IL-6, iNOS, and COX2. **(B)** Western blot band semi quantitative analysis. **(C)** Immunofluorescence staining and immunofluorescence intensity statistical analysis of TNF-α and iNOS in mouse joint synovium. scale bar: 100 μm. The data in each panel represent the means ± SD and p values were obtained by ANOVA t test, * p < 0.05, ** p < 0.01, *** p < 0.001, **** p < 0.0001.

We also investigate whether IL-19 can impede the inflammatory response of OA chondrocytes. Subsequently, western blot was performed to detect the protein expression of typical inflammatory factors in chondrocytes in different groups (**Figure 4A**). The results indicated that IL-19 treatment significantly reduced the protein expression levels of inflammatory factors TNF-α, IL-6, iNOS, and COX2 in IL-1β-treated chondrocytes (**Figure 4B**). Additionally, immunofluorescence analysis was carried out for mouse joint tissue sections. The results revealed strong positive signals for TNF-α and iNOS in the OA group, while treatment with IL-19 significantly suppressed the expression of these inflammatory mediators (**Figure 4C**). These findings suggest that IL-19 can alleviate the inflammatory response in OA mice.

### IL-19 inhibits cartilage matrix degradation and promotes matrix synthesis

**Figure 5:**
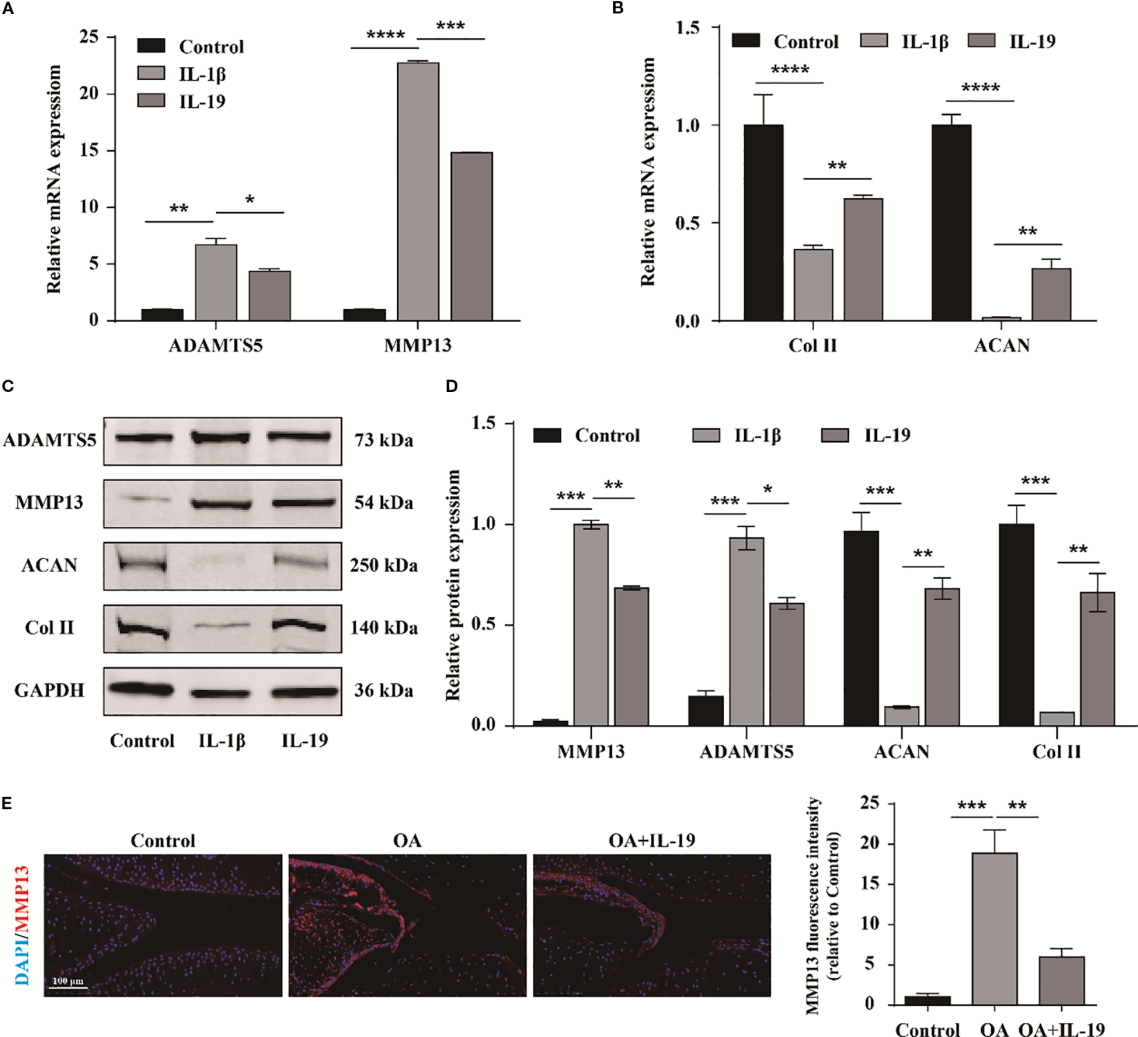
IL-19 inhibits cartilage matrix degradation and promotes matrix synthesis. **(A)** QPCR detection of mRNA expression of ADAMTS5 and MMP13. **(B)** QPCR detection of mRNA expression of ACAN and Col II. **(C)** Western blot imaging of ADAMTS5, MMP13, ACAN and Col II. **(D)** Western blot band semi quantitative analysis. **(E)** Immunofluorescence staining and immunofluorescence intensity statistical analysis of MMP13 in mouse joint synovium. scale bar: 100 μm. The data in each panel represent the means ± SD and p values were obtained by ANOVA test, * p < 0.05, ** p < 0.01, *** p < 0.001, **** p < 0.0001.

To further investigate the impact of IL-19 on OA phenotype, qPCR was applied to measure the expression of matrix degradation- related molecules ADAMTS5 and MMP13, as well as matrix synthesis-related molecules ACAN and Col II. The results demonstrated that IL-19 treatment significantly inhibited the expression of ADAMTS5 and MMP13 in IL-1β-treated chondrocytes (**Figure 5A**), and enhanced the expression of ACAN and Col II (**Figure 5B**). At the protein level, the results revealed consistent changes with the qPCR results for ADAMTS5, MMP13, ACAN, and Col II (**Figures 5C, D**). Furthermore, immunofluorescence analysis of MMP13 for mouse joint tissue sections exhibited that IL-19 treatment reduced the expression of MMP13 in OA mice (**Figure 5E**). Accordingly, our findings provide evidence that IL-19 decreases cartilage matrix degradation and enhance matrix synthesis in OA.

### The Hippo signaling pathway is involved in IL-19 mediated treatment of OA

**Figure 6:**
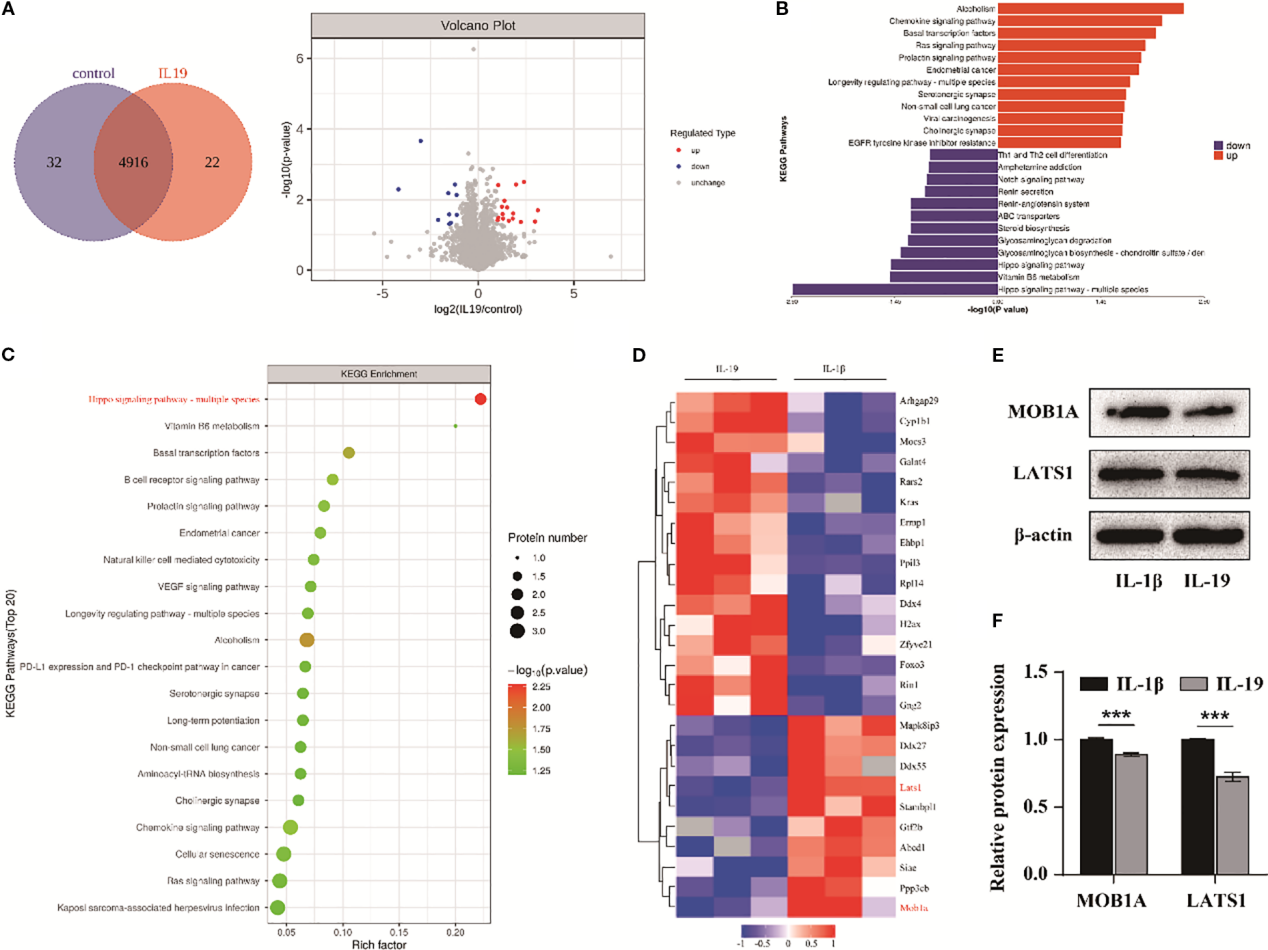
The Hippo signaling pathway is involved in IL-19 mediated treatment of OA. **(A)** Protein Venn diagram identified by proteomic sequencing and volcano diagram of differentially expressed proteins. **(B)** Enrichment butterfly diagram of pathways for upregulation and downregulation of differentially expressed proteins. **(C)** KEGG enrichment bubble diagram of differentially expressed proteins, with the horizontal axis representing the enrichment factor, bubble size representing the number of proteins, and bubble color representing the significance of enriched KEGG pathways. **(D)** Heatmap of significantly differentially expressed proteins. **(E)** Western blot detection of Hippo pathway related proteins. **(F)** Western blot band semi quantitative analysis. The data in each panel represent the means ± SD and p values were obtained by Student’s two-tailed unpaired t test, *** p < 0.001.

To investigate the underlying mechanisms triggered by IL-19 intervention, we conducted proteomic sequencing to identify differentially expressed proteins. A total of 4970 proteins were identified, with 32 proteins exclusively in the control group and 22 proteins unique to the IL-19 group (**Figure 6A**). The volcano plot format showed 16 significantly upregulated proteins in red and 10 significantly downregulated proteins in blue (**Figure 6A**). Subsequently, KEGG pathway enrichment analysis was conducted on the differentially expressed proteins. Notably, the Hippo signaling pathway exhibited the highest level of significance (**Figures 6B, C**). We further applied a hierarchical clustering algorithm to group and classify the differentially expressed proteins and presented significant results with fold change greater than 2 and p-value less than 0.05 in the form of heatmaps. It revealed that key components in the Hippo signaling pathway, MOB1A and LATS1, were significantly downregulated in the IL-19 group (**Figure 6D**). Western blot analysis was conducted to validate the changes in protein level of MOB1A and LATS1 and statistical results demonstrated that IL-19 intervention significantly reduced the protein level of both MOB1A and LATS1 (**Figures 6E, F**). The above results suggest that the Hippo signaling pathway may play a critical role in the inhibition of OA progression by IL-19.

### IL-19 inhibits the Hippo-YAP signaling pathway to slow down the progression of OA

**Figure 7:**
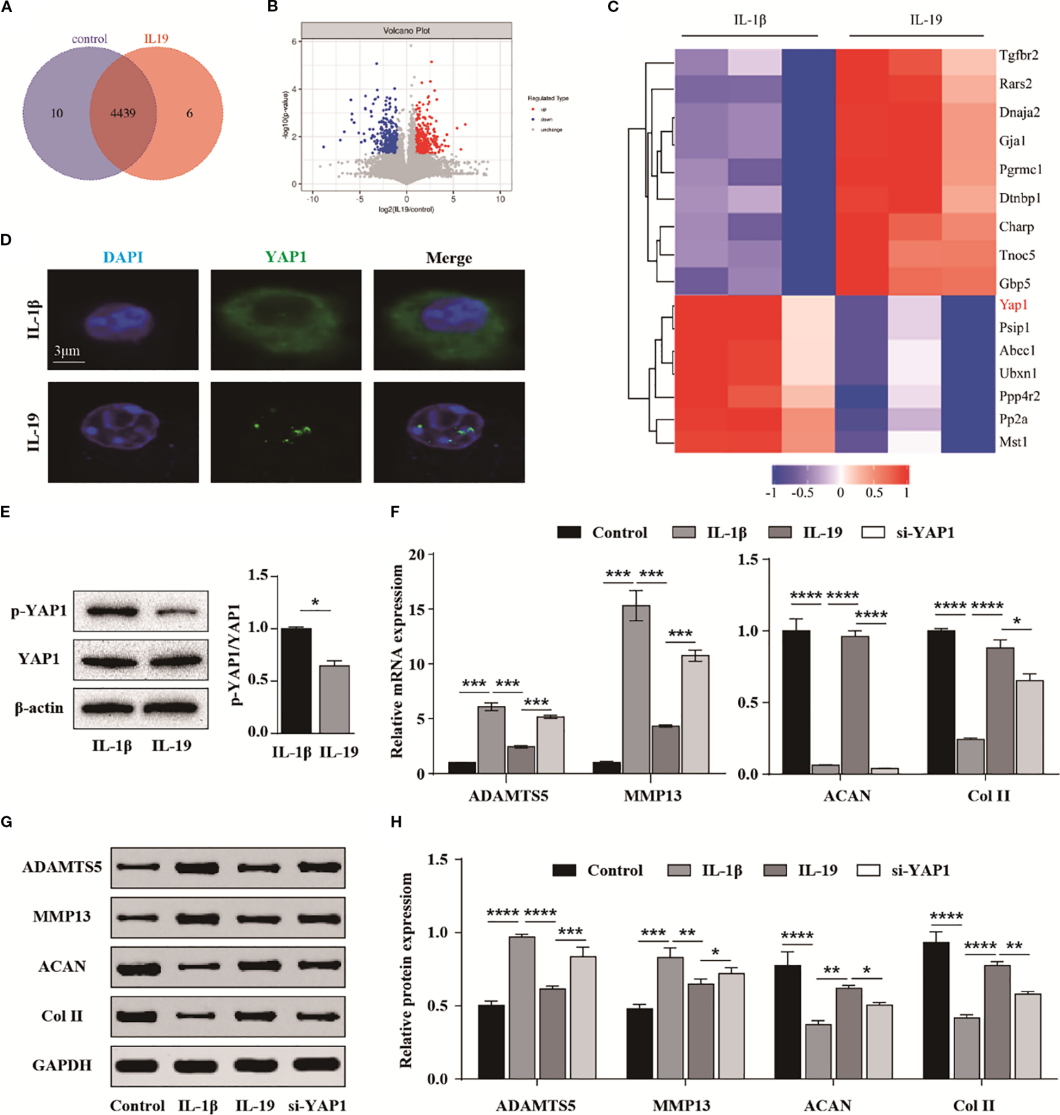
IL-19 inhibits the Hippo-YAP signaling pathway to slow down the progression of OA. **(A)** Venn diagram identified by phosphorylated proteomics sequencing. **(B)** Volcano diagram of differentially expressed phosphorylated peptide proteins. **(C)** Cluster analysis results of the top 16 most significantly different phosphorylated peptide proteins. **(D)** Immunofluorescence results for YAP1’s intracellular localization. **(E)** Western blot detection of phosphorylated YAP1 and semi quantitative analysis of p-YAP1/YAP1. **(F)** QPCR analysis of the effect of si-YAP1 on mRNA expression of ADAMTS5, MMP13, ACAN and Col II. **(G)** Western blot validation of the effect of si-YAP1 on protein expression of ADAMTS5, MMP13, ACAN and Col II. **(H)** Western blot band semi quantitative analysis. The data in each panel represent the means ± SD and p values were obtained by Student’s two-tailed unpaired t test **(E)** and ANOVA test **(F, H)**, * p < 0.05, ** p < 0.01, *** p < 0.001, **** p < 0.0001.

To explore the underlying mechanism, we further conducted phosphorylated proteomics sequencing. Totally, 4,455 proteins were identified, with 10 proteins exclusive to the control group and 6 proteins specific to the IL-19 group (**Figure 7A**). The volcano plot exhibited 514 significantly upregulated and 542 significantly downregulated phosphorylated peptide segments (**Figure 7B**). The heatmap presented the top 16 significantly altered phosphorylated peptide segments. Notably, we found that the phosphorylation of YAP1, a key component of the Hippo signaling pathway, was significantly decreased in the IL-19 group (**Figure 7C**). Then, immunofluorescence staining was performed and results revealed that IL-19 enhances the nuclear translocation of YAP1 (**Figure 7D**). Moreover, IL-19 intervention resulted in a significant reduction in the phosphorylation level of YAP1, which aligns with the findings from our phosphorylated proteomics sequencing (**Figure 7E**). To further verify whether IL-19 mitigates the progression of osteoarthritis through the Hippo-YAP signaling pathway, we transfected si-YAP1 into IL-19-treated chondrocytes. The qPCR results indicated that silencing of YAP1 partially reversed the inhibitory effects of IL-19 on ADAMTS5 and MMP13, and also diminished the promotion of ACAN and Col II expressions (**Figure 7F**). Subsequent verification at the protein level supported these findings (**Figure 7G**), and statistical analyses demonstrated consistent changes at the mRNA level (**Figure 7H**). The above results demonstrate that IL-19 alleviates IL-1β-induced progression of osteoarthritis by reducing the phosphorylation level of YAP1 and promoting its nuclear translocation.

### YAP1 targets Smad2 by interaction with TEAD1

**Figure 8:**
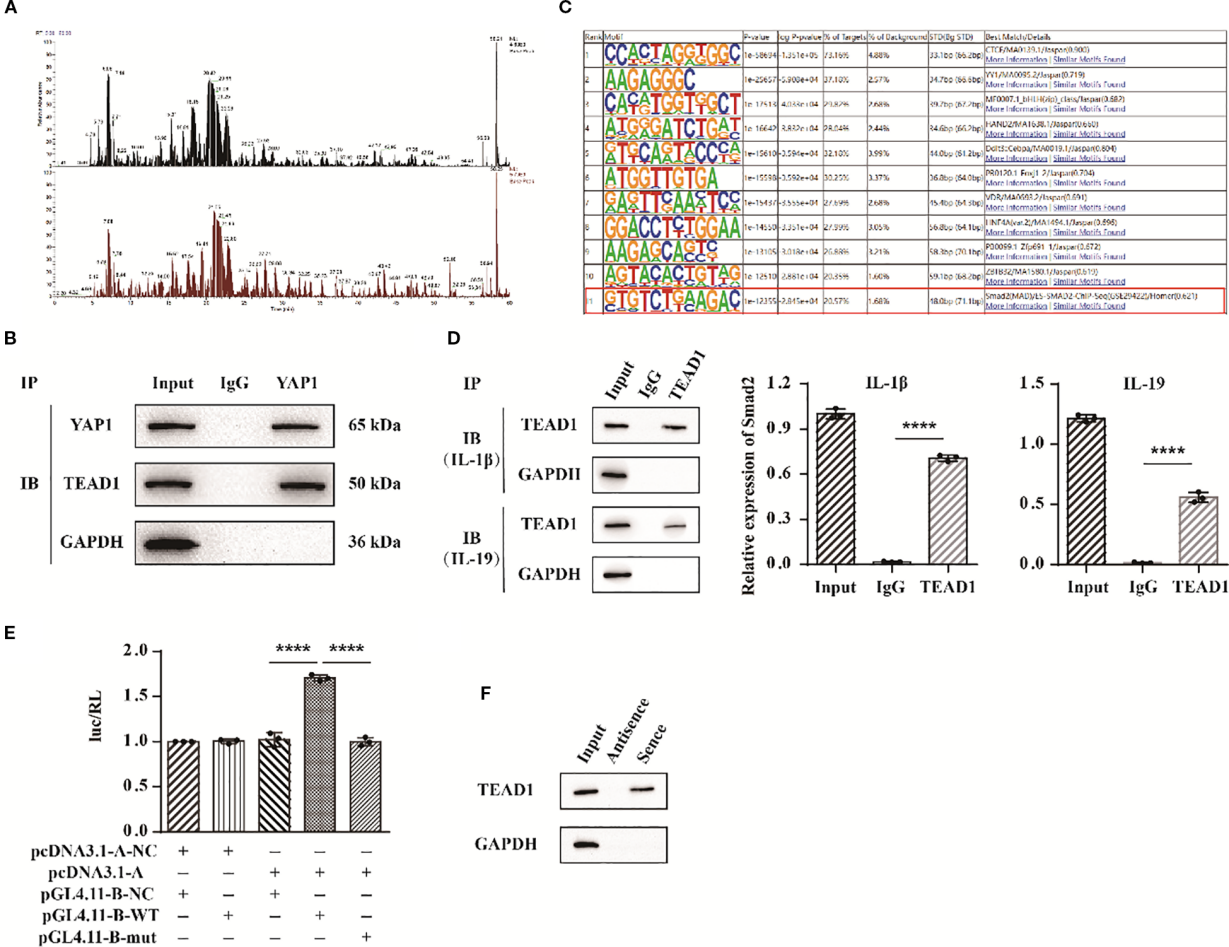
YAP1 targets Smad2 by interaction with TEAD1. **(A)** YAP1 protein mass spectrometry analysis. **(B)** Co-IP experimental bands of YAP1 and TEAD1. **(C)** ChIP-seq results of protein TEAD1. **(D)** ChIP-PCR results of Smad2. **(E)** Statistical chart of dual luciferase detection results of Smad2. **(F)** DNA pull down detection of TEAD1 protein bands. The data in each panel represent the means ± SD and p values were obtained by ANOVA test, **** p < 0.0001.

To further elucidate the role of YAP1, we employed protein mass spectrometry identification technology to identify potential target proteins of YAP1. Among the proteins interacting with YAP1, TEAD1 exhibited a high sequence coverage and the highest overall interaction score (**Figure 8A**). To validate the interaction between YAP1 and TEAD1, we conducted co-immunoprecipitation (Co-IP) experiments and results detected the presence of TEAD1 in the anti-YAP1 immunoprecipitation (**Figure 8B**). To investigate the underlying mechanism, ChIP-seq technology was applied to analyze the interaction between TEAD1 and targeted DNA. We specifically found the targeted gene Smad2 (**Figure 8C**). Subsequently, we performed ChIP-PCR to selectively detect Smad2 and confirmed the binding between the Smad2 promoter and TEAD1 (**Figure 8D**). Additionally, we co-transfected TEAD1 overexpression vectors along with wild-type (WT) and mutant (mut) dual luciferase vectors containing the Smad2 promoter sequences into 293T cells. Measurement of fluorescence intensity revealed that TEAD1 exerts a transcriptional regulatory effect on Smad2 (**Figure 8E**). Finally, we used biotin-labeled primers to isolate the promoter region sequence of Smad2 and incubated it with nuclear extracts. Through elution, we obtained the target DNA-protein complex and identified TEAD1 via western blot (**Figure 8F**). These findings suggest that the YAP1/TEAD1 complex targets the regulatory region of Smad2 to modulate downstream signaling pathways.

## Discussion

Osteoarthritis is an inflammatory disease that severely affects joint activity (21). In addition to mechanical stress, inflammation is an important factor in the progression of osteoarthritis (6, 22). As key immune cells, synovial macrophages play a critical role in both the symptomatology and structural progression of osteoarthritis (23, 24). Research has demonstrated that activated macrophages are regulated by various signaling pathways, including mTOR, NF-κB, JNK, and PI3K/Akt, and they polarize into different subtypes in osteoarthritis tissue (25, 26). In addition to autocrine interaction, the paracrine interaction between macrophages and chondrocytes also leads to cartilage damage through the inflammatory cytokines, growth factors, and MMPs (17, 27). It was reported that SCII immunomodulates M2 macrophages, shifts the local osteoarthritic microenvironment toward a pro-chondrogenic state, and promotes cartilage repair under inflammatory conditions (28). Therefore, this study focuses on the regulatory role of synovial macrophages in the environment of osteoarthritis. As an immune regulatory factor, IL-19 plays an important role in various inflammatory diseases relevant to macrophage activities (29). In patients with inflammatory bowel disease, macrophages exhibit significantly increased expression of IL-19 (30). Additionally, it has been reported that IL-19 can inhibit the progression of established atherosclerotic plaques by modulating both macrophage inflammation and cholesterol homeostasis (31). This study first explored the expression characteristics of IL-19 and found that it is highly expressed in osteoarthritis and co-localized with M2 macrophages.

The role of IL-19 in inflammatory response cannot be simply classified as either anti-inflammatory or pro-inflammatory; its immune regulatory functions remain inadequately understood, necessitating further detailed research. Increasing evidences suggest that IL19 interacts with the IL-20 receptor complex, comprising IL-20Rα and IL-20Rβ, which activates Signal Transducer and Activator of Transcription (STAT) 1 and STAT3 (12, 32, 33). This study found that the conditioned medium derived from M2 macrophages effectively inhibited matrix degradation and promoted matrix synthesis genes. IL-20Rβ receptor blockade reversed this phenomenon, demonstrating the important role of IL-19 in conditioned medium. Studies have also indicated that IL-19 stimulation leads monocytes to release IL-6 and TNF-α, ultimately promoting TNF-α-mediated apoptosis of cells (34). While it appears that monocytes do not express IL-20Rα, the IL-19 produced by these monocytes may facilitate signaling through paracrine pathways. Research has shown that IL-19, produced during the progression of rheumatoid arthritis (RA) and spondyloarthritis (SpA), acts as a pathogenic factor that enhances TNF-α levels in synovial tissue, along with increased expression of IL-1β, IL-6, and RANKL, thereby accelerating disease progression (35, 36). experimental findings indicate that the external application of IL-19 can inhibit apoptosis in synovial cells and stimulate IL-6 production by activating STAT3 (37). Our research demonstrated that exogenous addition of IL-19 can inhibit chondrocyte inflammation and apoptosis, and improve cartilage phenotype in osteoarthritis both *in vitro* and *in vivo*.

In addition, IL-19 plays an important role in various bone related diseases. It was demonstrated that IL-19 inhibits RANKL-induced NF-κB and p38 signaling pathways, thus disrupting osteoclast differentiation and mitigating the progression of osteoporosis (38). Other research has shown that lung cancer cells can induce osteoclasts to secrete IL-19 via GM-CSF, which in turn activates the IL-20Rβ receptor on tumor cells, ultimately promoting cell proliferation and facilitating their metastasis and colonization in bone (20). In cases of chronic non-bacterial osteomyelitis and chronic recurrent multifocal osteomyelitis, the absence of IL-10 and IL-19 has been linked to the assembly of NLRP3 inflammasomes, which results in elevated levels of inflammatory factors and disrupts the bone homeostasis (39). Therefore, elucidating the function and mechanism of IL-19 is of great value in revealing potential therapeutic targets for various diseases.

The KEGG enrichment results of proteomics showed that after IL-19 treatment, the Chemokine, Ras, and EGFR pathways were upregulated, while Hippo, ABC transporter, Notch pathways were downregulated. Studies have reported that key signaling pathways, including transforming growth factor-β (TGF-β), fibroblast growth factors (FGFs), mitogen-activated protein kinase (MAPK), hypoxia-related factors (HIF), Wnt/β-catenin, nuclear factor kappa B (NF-κB), and the Hippo/Yes-associated protein (YAP) pathway, are intricately linked to the cellular processes of chondrocytes (40, 41). Notably, Hippo/YAP signaling has been shown to play a crucial role in regulating cartilage physiology and is associated with the pathogenesis of OA (42, 43). This pathway is centered on a kinase cascade comprising mammalian Ste20-like kinase 1 (MST1), MST2, adaptor proteins Salvador 1 (SAV1), large tumor suppressor kinases 1 (LATS1) and 2 (LATS2), MOB kinase activators 1A (MOB1A) and 1B (MOB1B), as well as the transcriptional co-activators YAP and transcriptional co-activator with PDZ-binding motif (TAZ), which interact with the TEAD transcription factors (TEAD1–TEAD4) (44). Our study revealed that key components in the Hippo signaling pathway, MOB1A and LATS1, were significantly downregulated in the IL-19 group. YAP, serving as the principal effector in this signaling cascade, is directly phosphorylated by LATS1 and LATS2. When phosphorylated, YAP is retained in the cytoplasm where it undergoes rapid degradation through ubiquitination, thereby negating its growth-promoting and anti-apoptotic functions (40). Phosphorylated proteomics detected a decrease in YAP1 in the IL-19 group, and *in vitro* experiments demonstrated that IL-19 treatment inhibited YAP1 phosphorylation and promoted its nuclear translocation.

**Figure 9:**
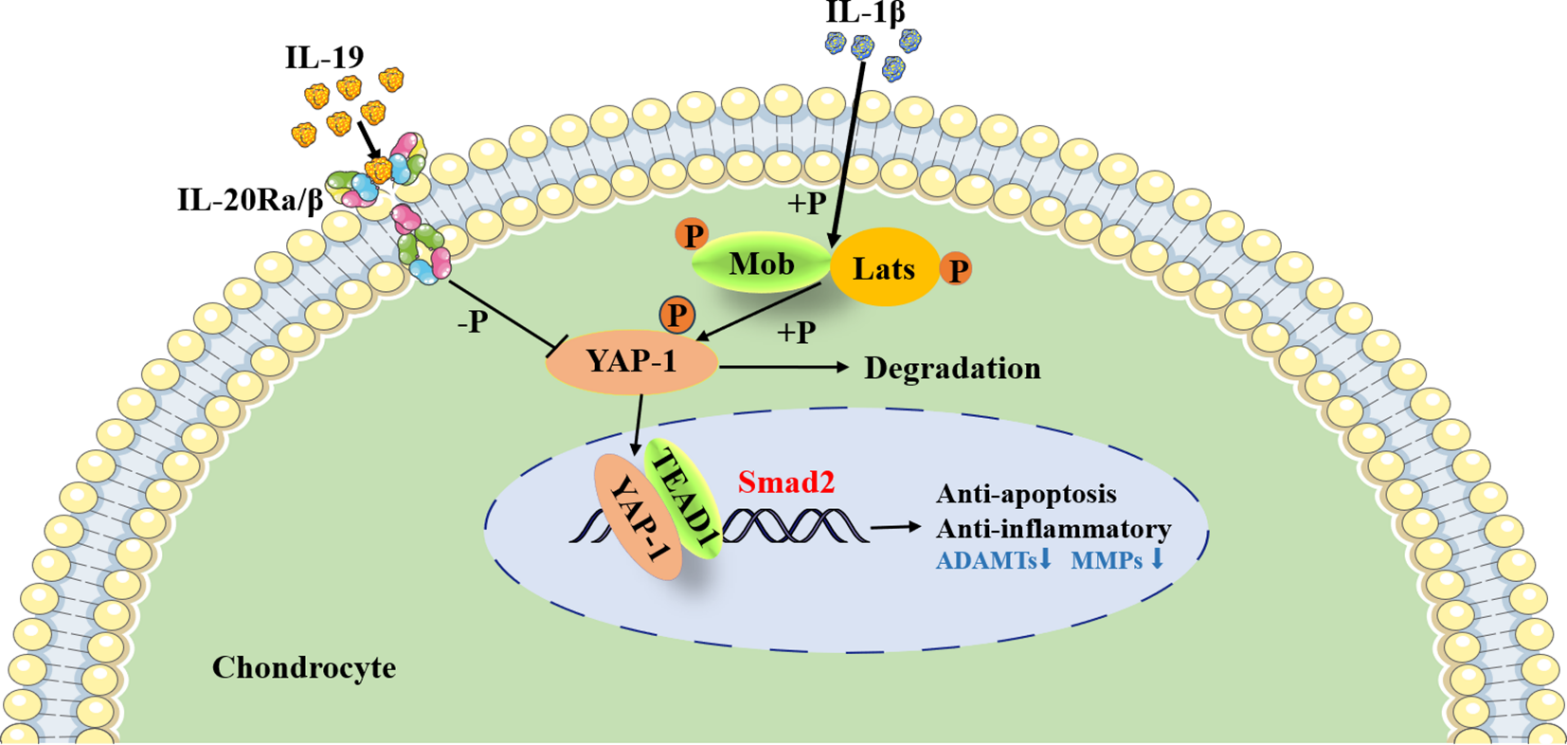
IL-19 alleviates IL-1β induced osteoarthritis via regulating YAP1 phosphorylation.

Furthermore, during cartilage development and differentiation, YAP has been identified as a negative regulator of chondrocyte differentiation; it upregulates Sox6 expression by binding to TEADs, thus promoting early chondrocyte proliferation (45). Moreover, YAP inhibits the expression of Col10a1, subsequently suppressing chondrocyte maturation through the regulation of the Wnt/β-catenin signaling pathway or interaction with Runx2 (45). YAP also plays a vital role in preserving the phenotype of chondrocytes; increasing fluid shear stress has been observed to enhance YAP expression, ultimately resulting in the dedifferentiation of chondrocytes and loss of their characteristic traits (46). Conversely, hypoxia triggers the activation of HIF-1α, which in turn promotes YAP-mediated maintenance of the cartilage phenotype (47).. Research has shown that the activity of the Hippo-YAP pathway changes with the progression of OA, yet its precise role in OA remains unclear. In an OA mouse model induced by medial meniscus injury, it was observed that nuclear expression of YAP significantly increased compared to normal mice four weeks post-surgery (48). On the contrary, Deng et al. reported a decrease in YAP expression in human OA cartilage, with levels declining in correlation with the severity of OA (45). Similar results were noted in mice with OA induced by ACLT (49). Furthermore, it was found that the pro-inflammatory cytokines strongly trigger the phosphorylation of MST1 and LATS1, which promotes the degradation of YAP in the cytoplasm of mouse chondrocytes (50). In this study, we found that silencing YAP1 interfered with the regulatory effect of IL-19 on cartilage phenotype, further demonstrating its targeted transcriptional regulation of smad2 through synergistic action with TEAD1 (**Figure 9**).

In conclusion, this study reveals that IL-19, secreted by M2 synovial macrophages in OA microenvironment, inhibits the inflammatory response and apoptosis of chondrocytes, and mitigates the destruction of the joints through paracrine signaling, providing new insights into the role of macrophages in the progression of OA.

## Data Availability

The original contributions presented in the study are included in the article/supplementary material. Further inquiries can be directed to the corresponding authors.
